# Should we breathe more like dogs when overheated? A perspective from acid–base balance

**DOI:** 10.1113/EP093499

**Published:** 2025-12-13

**Authors:** Akira Katagiri, Naoto Fujii

**Affiliations:** ^1^ Institute of Health and Sport Sciences University of Tsukuba Tsukuba City Ibaraki Japan; ^2^ Advanced Research Initiative for Human High Performance (ARIHHP) University of Tsukuba Tsukuba City Ibaraki Japan

**Keywords:** heat stroke, respiration, thermoregulation

1

Pulmonary ventilation is tightly regulated to match metabolic demands, specifically O_2_ consumption and CO_2_ production, maintaining arterial blood gases. However, when humans are passively heated, hyperventilation occurs as core temperature rises, even at rest where metabolism remains almost constant. This response leads to hypocapnia, which is accompanied by blood and cerebral intra‐ and extracellular alkalosis, thereby disturbing acid–base homeostasis. Notably, this response appears distinct from the tachypnoeic panting observed in some acutely heat‐stressed animals, such as dogs, in which respiratory frequency markedly increases while tidal volume decreases, resulting in minimal change in alveolar ventilation (Maskrey & Jennings, 1985; Robertshaw, 2006). This breathing pattern enhances evaporative and conductive heat dissipation from the airways but does not induce blood alkalosis. Thus, the physiological significance of the breathing response during heat stress appears to differ between panting animals and humans. It remains unclear whether hyperthermia‐induced hyperventilation in humans represents an adaptive physiological mechanism aimed at modulating blood acid–base balance. Accordingly, the optimal approach to hyperthermia‐induced hyperventilation, whether it should be augmented or attenuated, remains to be elucidated. If hyperthermia‐induced hyperventilation serves to mediate blood alkalosis for maintaining homeostasis, then enhancing blood alkalosis would be expected to attenuate this response, because alkalosis has already developed, and further increases in ventilation would be unnecessary. Conversely, inducing additional blood acidosis would be expected to enhance the hyperventilation.

In this context, in the study by Soo et al. (2025), published in this issue of *Experimental Physiology*, the authors experimentally manipulated baseline blood acid–base balance before passive heating and investigated its effect on the core temperature‐dependent ventilatory response in resting humans. They demonstrated that prior blood alkalosis achieved by sodium bicarbonate ingestion did not reduce absolute ventilation but reduced the ventilatory equivalent for CO_2_ production compared with control conditions throughout the passive heating, thereby increasing end‐tidal partial pressure of CO_2_ (an index of arterial partial pressure of CO_2_). Similar results were also obtained in our recent work (Katagiri et al., 2024). Notably, Soo et al. (2025) extended our previous work by demonstrating that blood acidosis mediated by ammonium chloride ingestion increased absolute ventilation and decreased end‐tidal partial pressure of CO_2_ compared with control conditions throughout the heating. Consequently, the authors concluded that the magnitude and characteristics of the ventilatory response during hyperthermia were influenced by prior alkalaemia or acidosis. However, the influence of blood alkalosis or acidosis on ventilation occurred before elevation in core temperature, and the magnitude of altered ventilation was similar during heating. This is supported by their results, which showed a significant main effect of the acid–base conditions, without a significant interaction between core temperature and acid–base status. We therefore think that although blood alkalosis or acidosis can influence level of breathing for acid–base balance, it does not influence hyperthermia‐induced hyperventilation. Based on the study by Soo et al. (2025) and our previous work (Katagiri et al., 2024), we think that hyperthermia‐induced hyperventilation is unlikely to serve a purposeful role in blood acid–base regulation.

The physiological significance of hyperthermia‐induced hyperventilation has been debated in terms of thermal homeostasis. Increased breathing can theoretically increase respiratory evaporative and conductive heat loss and is potentially important in thermoregulation, such as selective brain cooling analogous to panting in some animals (White et al., 2011). However, it should be noted that the vascular anatomy of the head in panting species differs from that of humans (Cabanac, 1986). In panting animals, such as dogs, heat is dissipated through the nasal and oral cavities (Hellstrom & Hammel, 1967), and the cooled venous blood drains into the cavernous sinus, where it interacts with the internal carotid artery (i.e., rete mirabile) (Cabanac, 1986; Robertshaw, 2006). This arrangement facilitates countercurrent heat exchange and enables effective selective brain cooling. In contrast, humans lack a comparable rete, suggesting that this brain‐cooling mechanism might not exist in humans. Notably, hypocapnia secondary to hyperthermia‐induced hyperventilation reduces cerebral blood flow, potentially impairing cerebral heat removal (Nybo et al., 2002). Moreover, exacerbated breathing can cause the perception of breathlessness, thereby increasing psychological stress. Thus, hyperthermia‐induced hyperventilation definitely increases physiological and psychological stress. Furthermore, hyperthermia‐induced hyperventilation shows considerable inter‐individual variability in healthy young individuals. In some individuals, hyperventilation begins as soon as core temperature rises, whereas in others it does not occur even when core temperature exceeds 39°C. If hyperthermia‐induced hyperventilation serves a homeostatic function, this response would be expected to occur more consistently. Therefore, it does not appear to represent an adaptive physiological mechanism, including thermal regulation or blood acid–base balance.

Integrating the new findings by Soo et al. (2025), at present, hyperthermia‐induced hyperventilation does not appear to be aimed primarily at mediating blood alkalosis. Moreover, augmenting hyperthermia‐induced hyperventilation and the resultant hypocapnia is unlikely to be beneficial from the perspective of brain temperature regulation, given that hypocapnia decreases cerebral blood flow. Should we breathe more when heated? Answer is perhaps no. Rather, from this viewpoint, we propose that strategies aimed at attenuating, preventing or not exacerbating hyperthermia‐induced hyperventilation might decrease physiological and psychological stress, thereby potentially enhancing human safety and tolerance during heat stress. Given that hyperthermia‐induced hyperventilation is closely associated with core temperature, the most effective interventions would be those that directly reduce core temperature. These include external cooling methods, such as water immersion, internal cooling through ingestion of cold fluids or ice slurry (Katagiri et al., 2025), and heat acclimatization. Voluntary control of breathing might also be beneficial. Additionally, avoiding ventilatory stimulants, such as caffeine, might help to prevent further exacerbation of the response. Future research is expected to expand these intervention options and clarify the underlying mechanisms by which they exert beneficial effects.

## AUTHOR CONTRIBUTIONS

Akira Katagiri and Naoto Fujii drafted the manuscript, edited and revised manuscript and approved the final version.

## CONFLICT OF INTEREST

None declared.

## FUNDING INFORMATION

None.
